# Effects of Reliability and Global Context on Explicit and Implicit Measures of Sensed Hand Position in Cursor-Control Tasks

**DOI:** 10.3389/fpsyg.2015.02056

**Published:** 2016-01-12

**Authors:** Miya K. Rand, Herbert Heuer

**Affiliations:** IfADo-Leibniz Research Centre for Working Environment and Human FactorsDortmund, Germany

**Keywords:** aiming, tool use, sensory coupling, implicit measure, explicit measure

## Abstract

In a cursor-control task in which the motion of the cursor is rotated randomly relative to the movement of the hand, the sensed directions of hand and cursor are mutually biased. In our previous study, we used implicit and explicit measures of the bias of sensed hand direction toward the direction of the cursor and found different characteristics. The present study serves to explore further differences and commonalities of these measures. In Experiment 1, we examined the effects of different relative reliabilities of visual and proprioceptive information on the explicitly and implicitly assessed bias of sensed hand direction. In two conditions, participants made an aiming movement and returned to the start position immediately or after a delay of 6 s during which the cursor was no longer visible. The unimodal proprioceptive information on final hand position in the delayed condition served to increase its relative reliability. As a result, the bias of sensed hand direction toward the direction of the cursor was reduced for the explicit measure, with a complementary increase of the bias of sensed cursor direction, but unchanged for the implicit measure. In Experiment 2, we examined the influence of global context, specifically of the across-trial sequence of judgments of hand and cursor direction. Both explicitly and implicitly assessed biases of sensed hand direction did not significantly differ between the alternated condition (trial-to-trial alternations of judgments of hand and cursor direction) and the blocked condition (judgments of hand or cursor directions in all trials). They both substantially decreased from the alternated to the randomized condition (random sequence of judgments of hand and cursor direction), without a complementary increase of the bias of sensed cursor direction. We conclude that our explicit and implicit measures are equally sensitive to variations of coupling strength as induced by the variation of global context in Experiment 2, but are differently sensitive to variations of the relative reliabilities as induced by our additional unimodal proprioceptive information in Experiment 1.

## Introduction

In many tasks, the position of ones’ own hand can be sensed visually and proprioceptively, and both sources of information are efficiently combined to obtain a single percept of the hand at a certain position (Van Beers et al., 1999). In the task of controlling the position of a cursor on a computer monitor, or more generally in tool use, visual and proprioceptive information typically are no longer fully redundant in that visual information refers to the position of the cursor and proprioceptive information to the position of the hand. Thus, the two sensory modalities refer to different objects in different planes of motion. However, there is a systematic relation between the movements of the hand and the motions of the cursor, and this relation could be sufficient to prompt sensory coupling (Ernst, 2006; Parise et al., 2012). In fact, we observed such coupling in a cursor-control task (Rand and Heuer, 2013): hand and cursor were clearly distinct percepts, but when discrepancies between their directions of movement were introduced, the sensed directions became mutually attracted. This mutual attraction was asymmetric in that the bias of sensed hand direction toward the direction of the cursor was stronger than the bias of sensed cursor direction toward the direction of the hand.

In the present experiments, we explore differences and commonalities between two measures of the bias of sensed hand direction toward the direction of the cursor in a cursor-control task. The first measure is based on explicit judgments. After an aiming movement in a particular direction and the return to the start position, participants move their hand along a circular path to its remembered position at the end of aiming. These judgments are psychophysical judgments of the perceived and remembered hand direction that participants are consciously aware of. The deviation of these judgments from the physical direction of the hand serves as an explicit measure of the bias of sensed hand direction toward the direction of the cursor. For short, we refer to this measure as explicit measure of hand direction.

The second measure of the bias of sensed hand direction toward the direction of the cursor is implicit in that it is based on the movements of the participants without them being aware of judging the direction of their hand. As detailed in the Methods section, this measure is the angle between the aiming movement and the return movement back to the start position. Its rationale is based on the observation that discrepancies between sensed and physical locations of the hand give rise to systematic errors in subsequent movements (Bock and Eckmiller, 1986; Rossetti et al., 1995; Heuer and Sangals, 1998; Holmes et al., 2004; Holmes and Spence, 2005; Heuer and Sülzenbrück, 2012). For short, we refer to this measure as implicit measure of hand direction.

Our exploration of explicit and implicit measures of hand direction is motivated by some findings on visuo-motor adaptation. Visuo-motor adaptation involves both implicit and explicit components (Mazzoni and Krakauer, 2006; Heuer and Hegele, 2011; Heuer and Sülzenbrück, 2013; Taylor et al., 2014; Rand and Rentsch, 2015). Whereas the explicit component is reduced at older adult age, the implicit component is generally stable at least until about age 65 (see Heuer et al., 2013, for review). The age-related change of the explicit component turned out to be associated with an age-related decline of the discrimination of the directions of hand movement and concurrent cursor motion (Rand et al., 2013), which again was associated with an age-related increase of the explicit measure of the bias of the judged direction of hand movement toward the direction of cursor motion (Rand and Heuer, 2013). The implicit measure of the bias, in contrast, remained stable across the age range studied, consistent with the absence of an age-related decline of the implicit component of visuo-motor adaptation.

In addition to the contrasting age-related changes, Rand and Heuer (2013) observed other differences between the explicit and implicit measures of sensed hand direction. First, the explicitly measured bias was stronger than the implicitly measured bias. Second, the explicit measure had considerably larger intra-individual variability than the implicit measure. Third, sequential effects of the psychophysical judgment required in each trial (judgment of cursor or hand direction) were different for the explicit and implicit measures of hand direction. Finally, the individual explicitly and implicitly measured biases of sensed hand direction were uncorrelated. The differences between the two measures of bias and the lack of correlation suggest that they are based on different combinations of visual and proprioceptive information (cf. Knill, 2005; Sober and Sabes, 2005). In terms of a dichotomy, the explicit and implicit measures might tap different representations of hand position that serve different purposes – cognition and motor control, respectively – and that are differently accessible by conscious awareness (cf. Head and Holmes, 1911; Paillard, 1991; Dijkerman and de Haan, 2007; de Vignemont, 2010).

At present, the origin of the differences between the explicit and implicit measures of sensed hand direction is not yet sufficiently clear. They might indeed result from different representations of the position of the hand, which combine proprioceptive information related to the hand and visual information related to the cursor with different relative weights. However, they might also result from a single representation of the position of the hand, with the differences between the two measures being caused by additional factors. As an example, consider the smaller implicit than explicit bias measures. For a certain discrepancy between sensed and physical hand positions at the end of the aiming movements, as indicated by the explicit measure, the directional error of the subsequent return movements might not reflect the full discrepancy, but only a part of it (cf. Rossetti et al., 1995). This would be the case if the return movement would not only be coded in terms of amplitude and direction (e.g., Gordon et al., 1994; Rossetti et al., 1995; Vindras and Viviani, 1998, 2002; Wang and Sainburg, 2005), but also in terms of the final position (e.g., Kelso and Holt, 1980; Schmidt and McGown, 1980; Bizzi et al., 1984) or posture (Rosenbaum et al., 1995). For motor memory in particular, both an influence of amplitude and end position has been shown (e.g., Laabs, 1974; Gundry, 1975; Jaric et al., 1994). An influence of end position coding could account for the smaller implicit than explicit bias measures. For other differences between the two measures, however, accounts that posit only a single representation of hand position and different ways of making use of it are not that obvious.

The present study was designed to explore further potential differences between the implicitly and explicitly assessed biases of hand direction toward the direction of the cursor in a cursor-control task. Such additional differences would bolster the hypothesis that the implicit and explicit measures tap different representations of hand position. In particular, we examined the effects of varying the relative reliabilities of visual and proprioceptive information (Experiment 1) and the influence of global context, specifically the across-trials arrangement of judgments of hand and cursor direction (Experiment 2).

## Experiment 1

Models of sensory integration typically posit a weighted average of different sensory signals, with the weights being adjusted to the reliabilities of the individual signals. This type of integration serves to minimize the variance of the multimodal estimates (cf. Ernst and Bülthoff, 2004; Cheng et al., 2007). However, as pointed out by Sober and Sabes (2005; cf. Greenwald and Knill, 2009), minimum-variance models are incomplete in that the weights of the combined sensory signals do not only depend on the reliabilities, but also on task-specific factors.

Regarding reliabilitiesin the case of the cursor-control task considered here, the visual signal related to the direction of cursor motion is probably more reliable than the proprioceptive signal related to the direction of hand movement. In general, dominance of visual information over sensory inputs of other modalities is fairly well established (e.g., Ernst and Banks, 2002; Besson et al., 2010). With the cursor-control task, Rand and Heuer (2013) observed smaller variability of the judged direction of the cursor than of the judged direction of the hand, consistent with higher reliability of visual than of proprioceptive information. Regarding the specifics of cursor-control tasks, attention tends to be focused on the visual information (Collins et al., 2008; Reed et al., 2010), and conscious awareness of the position of the hand is only poor (Müsseler and Sutter, 2009). In fact, there is evidence that proprioceptive input is functionally neglected during tool use (Heuer and Rapp, 2012), for example by inhibiting its access to the somatosensory cortex (Bernier et al., 2009). Taken together, both the reliabilities and the task specifics lead one to expect a stronger weight of the visual modality so that the bias of the sensed direction of the cursor toward the direction of the hand should be smaller than the bias of the sensed direction of the hand toward the direction of the cursor – which is consistent with the asymmetry observed with explicit measures of cursor and hand direction.

In the first experiment, we varied the relative reliabilities of visual and proprioceptive information in a particular way. In the “immediate” condition, an aiming movement to a remembered target was performed, followed immediately by the return movement to the remembered start position and a subsequent judgment of cursor or hand direction at the end of aiming. In the “delayed” condition, the start of the return movement was delayed by about 6 s. During the delay, the cursor was no longer visible, but the hand remained in the final position of the aiming movement. Thus, visual information on the final position of the cursor in the delayed condition was basically identical to that in the immediate condition, except for some decay because of the delay, but proprioceptive information on the final position of the hand was presented for a longer duration and without concurrent visual information on cursor position. Thereby the relative reliability of proprioceptive information on final hand position – relative to the visual information on final cursor position – was enhanced as compared with the immediate condition. If the weights of sensory coupling in the cursor-control task depended on the reliabilities of proprioceptive and visual information, the bias of sensed hand direction toward the direction of cursor motion should be reduced in the delayed condition as compared with the immediate one (cf. Bellan et al., 2015), and the bias of sensed cursor direction toward the direction of hand movement should be increased.

Our main interest was in possibly different effects of the variation of the relative reliabilities of proprioceptive and visual information on the explicitly- and implicitly measured bias of sensed hand direction. Such different effects are suggested by the following consideration. On the one hand, we are not continuously consciously aware of the position of our hand, but only when it is required, e.g., by the request to provide a psychophysical judgment. On the other hand, we produce a rather continuous stream of hand movements, and this likely requires a rather continuously updated representation of hand position. From this potential difference, one could expect that sensory coupling might occur at different times – at selected discrete points in time when hand-position information is required for judgments and thus explicit measures of hand direction, but rather continuously when hand-position information is required for movement control and thus implicit measures.

Our manipulation of the relative reliabilities of visual and proprioceptive information was restricted to the end of aiming. Throughout the aiming movement, except at its end, the difference between the reliabilities was the same in the immediate and the delayed condition. Only after the end position had been reached, the reliability of proprioceptive information was boosted by its unimodal presentation in the delayed condition. Even though proprioceptive information can decay somewhat with a static hand position after the end position has been reached (cf. Paillard and Brouchon, 1968; Craske and Crawshaw, 1975; Wann and Ibrahim, 1992), the decay of the no longer present visual information should have been stronger (cf. Bellan et al., 2015). Thus, when sensory coupling takes place just before a psychophysical judgment of hand direction is given, it should reflect the different relative reliabilities in the two conditions. In contrast, when sensory coupling takes place continuously as long as bimodal information is accessible, it might not or only little be affected by the additional unimodal proprioceptive information in the delayed condition. This should be the more so as proprioceptive information during the movement is typically more reliable than after the final position has been reached (cf. Paillard and Brouchon, 1968; Craske and Crawshaw, 1975; Wann and Ibrahim, 1992), probably because of the contribution of outflow information (corollary-discharge, Sperry, 1950, or efference-copy, Von Holst and Mittelstaedt, 1950). Therefore, we hypothesized that the explicitly assessed bias of sensed hand position should be reduced by the enhanced proprioceptive information after the end of the movement (accompanied by an increased explicitly assessed bias of sensed cursor direction), but that the implicitly assessed bias of hand direction should not be reduced or less than the explicit bias. Note that in the delayed condition, the interval during which the start position had to be remembered was also increased relative to the immediate condition; this could have resulted in a larger variability of the direction of the 3rd stroke relative to the 2nd stroke, but not in a change of the mean, and thus the implicitly assessed bias.

### Materials and Methods

#### Participants

Twenty-eight healthy right-handed participants (mean ± SD: 25.1 ± 2.9 years; 14 males and 14 females) signed informed consent. The study was conducted in accordance with the Declaration of Helsinki and with the general approval by the ethics committee of the Leibniz Research Centre for Working Environment and Human Factors.

#### Apparatus and Procedure

The experimental setting and procedures were similar to those of Rand and Heuer (2013). In brief, seated participants held a stylus with their right hand and made three-stroke movements on a digitizer (Wacom Intuos 4 XL, 133 Hz sampling rate). They faced a monitor which was covered by a large black circular screen with a semi-circular window (32 cm in diameter) in its center. A first target (T1, 1.4 cm in diameter) was located in that center, and the start position (SP, 1.2 cm in diameter) was located 3 cm below T1. A second target (T2, 1 cm in diameter) was presented at pseudo random locations, ranging from -60° to +60° relative to the central location, on an invisible circle with a radius of 15 cm around T1. An opaque board placed above the participants’ arm blocked their direct view of the hand.

**FIGURE 1:**
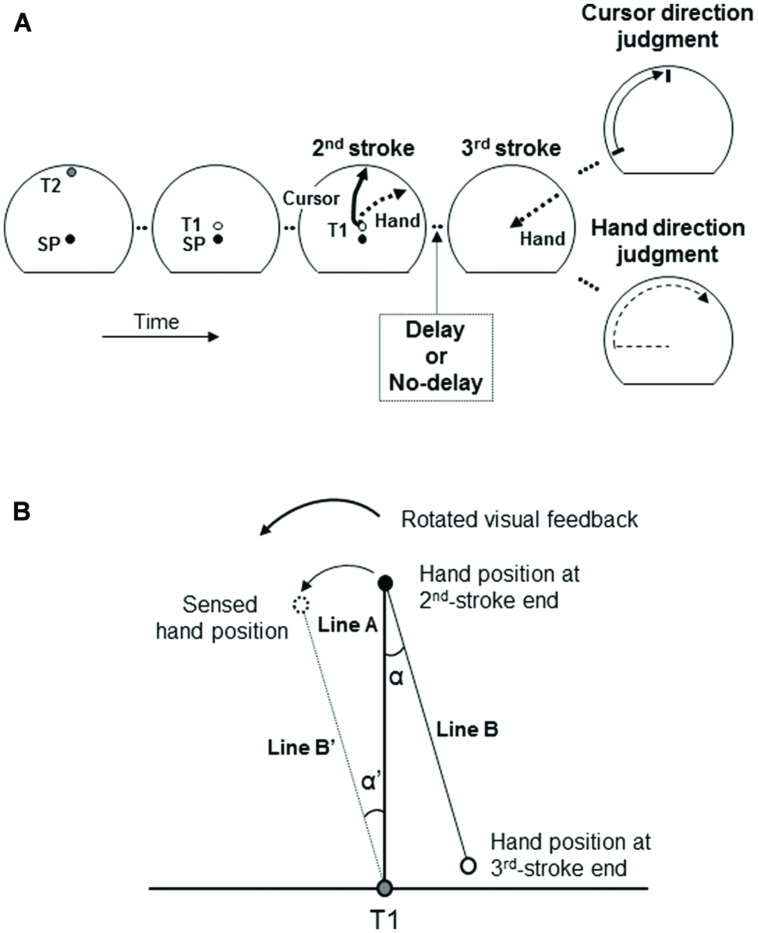
**Behavioral task of a three-stroke movement and judgments of hand and cursor directions **(A)**.** SP, T1 and T2 refer to a starting position, a first target, and a second target, respectively. The visual–feedback of the 2nd-stroke is rotated and displayed simultaneously with hand movements. After the 2nd-stroke, the participants make a return movement without the visual–feedback as the 3rd-stroke, and subsequently make an explicit judgment regarding the hand or cursor direction. Arrows with dashed line refer to hand movements (not the visual–feedback). A delay of 6 s is inserted after the 2nd-stroke in the delayed condition or not inserted in the immediate condition. **(B)** Implicit measure of hand direction. The directional deviation α’ of the sensed hand position (dotted outline circle) from the physical one (black circle) at the end of the 2nd-stroke is estimated from the directional deviation α of the hand position at the end of the 3rd-stroke (solid outline circle) from its remembered target (T1, gray circle).

At the beginning of each trial, participants were guided to the SP by arrows shown on the monitor. One second after the stylus was in the SP, T2 appeared for 1 s (**Figure 1A**, 1st panel). Subsequently, T1 appeared. After a delay of 0.5 s, an auditory go-signal was delivered. The participants then made three-stroke movements from the SP to T1 (1st stroke), to T2 (2nd stroke), and back to T1 (3rd stroke) at a comfortable speed. When the 1st stroke was made to T1, this target disappeared. Then, the participants made the 2nd stroke to the remembered T2 (**Figure 1A**, 3rd panel) until the movement was stopped by a stopper ring, a semi-circular plastic ring with a radius of 15 cm around T1 placed on the digitizer’s surface. Afterward, they made a return movement (3rd stroke) back to the remembered T1 location (**Figure 1A**, 4th panel).

The participants made the 1st and 2nd strokes with concurrent visual feedback provided by a cursor on the monitor, but the 3rd strokes without visual–feedback. Only during the 2nd stroke, the direction of cursor motion was rotated relative to the direction of hand movement by a randomly chosen angle out of 6 (clockwise [CW] direction: -30°, -18°, -6°; counter-clockwise [CCW] direction: 6°, 18°, 30°), with the constraint of equal frequencies of the 6 angles. The randomization of rotation angles served to prevent adaptation to a visuo-motor rotation.

In the delayed condition, a delay of 6 s was inserted after the end of the 2nd stroke, whereas there was no such delay in the immediate condition (**Figure 1A**). During the delay the cursor was invisible. A short beep at the end of the delay in the delayed condition and at the end of the 2nd stroke in the immediate condition signaled the participants to start the 3rd stroke.

One second after completing the 3rd stroke, participants were asked to judge the direction of either the hand or the cursor at the end of the 2nd stroke. For the judgment of cursor direction (**Figure 1A**, 5th panel, top), a short line (2 cm) was displayed. It marked the peripheral end of a radial line from T1 to the circumference of the invisible ring of 15 cm diameter centered at T1. The radial line, and thus its visible peripheral end, moved at a constant speed CCW or CW, beginning at a start position 102° to the right or left of the vertical. The participant instructed the examiner to stop and finely adjust (back and/or forth) the line to the direction that matched the judged direction of the cursor at the end of the 2nd stroke. For the judgment of hand direction (**Figure 1A**, 5th panel, bottom), the participant moved the pen without visual–feedback from the right (or left) lower corner of the stopper ring CCW (or CW) along the ring and stopped where he/she judged the hand direction to match the direction of the hand at the end of the 2nd stroke. Judged direction (hand or cursor direction) and direction of line or hand movement during the judgment (CW or CCW) were randomized across trials, again with the constraint of equal frequencies.

Judgments of cursor and hand direction were obtained with different responses, i.e., verbal responses to guide the experimenter in matching the position of a visual marker to the remembered position of the cursor, and manual responses to match the sensed position of the hand to the remembered position at the end of the 2nd stroke. Although different from each other, however, the responses which provided the explicit measures were closely related to the judged objects: the position of a visual marker had to be matched to the previous position of the visually perceived cursor, and the position of the proprioceptively perceived hand had to be matched to the previous position of the proprioceptively perceived hand. Moreover, judgments were obtained in different coordinate systems for the cursor and the hand, but in the same coordinate systems in which the respective judged objects were localized, defined by the computer screen in a vertical plane and the manual workspace in a horizontal plane. Note that both the motion of the visual marker and the movement of the hand during the judgments differed from cursor motion and hand movement, respectively, during the 2nd stroke. Therefore, only positions could be matched, but not directions and/or amplitudes of movements.

Before data recording, there were 10 familiarization trials that included the procedure without the visual–feedback rotation and/or without the judgment and four practice trials that included all the procedure (one trial for each combination of delayed vs. immediate and hand vs. cursor judgment). The experiment consisted of four sets of trials, each of which included 1 warm-up trial and 36 experimental trials (six trials for each of the six angular rotations). Across the four sets, the immediate and the delayed condition alternated. Across participants, the order of the immediate and delayed conditions was counterbalanced. A total of 72 experimental trials were recorded and analyzed for each condition.

#### Data Analysis

The angular deviation of the judged hand or cursor direction from the actual hand or cursor direction at the end of the 2nd stroke was measured in each trial (the CCW direction had a positive sign). Individual means and standard deviations of the angular deviations were computed for each judged direction (cursor or hand), each delay condition, and each visual–feedback rotation. The influence of the rotated visual–feedback on the deviations of the judged from the actual hand or cursor directions was assessed by the slope of a linear regression of the angular deviation (dependent variable) on the visual–feedback rotation (independent variable), computed for each participant, delay condition, and judged direction (cursor or hand). These slope parameters specify the strength of sensory coupling in terms of the biases of the judgments of hand and cursor directions in degree per degree of the visual–feedback rotation. We used them as explicit measures of the biases of sensed hand direction toward the direction of the cursor and of sensed cursor direction toward the direction of the hand.

As implicit measure of the bias of sensed hand direction toward the direction of the cursor, we computed the angular deviation of the direction of the 3rd stroke from the direction of the 2nd stroke in each trial, that is, α’ = α in **Figure 1B.** This measure exploits the existence of error propagation in successive aiming movements (Bock and Eckmiller, 1986; Heuer and Sangals, 1998; Heuer and Sülzenbrück, 2012), in particular the propagation of errors that originate from visually induced deviations between the physical and the sensed position of the hand (Rossetti et al., 1995; Holmes et al., 2004; Holmes and Spence, 2005). In our case, the visually induced angular deviations of the sensed position of the hand from the actual one (angle α’ in **Figure 1B**) occur in the 2nd stroke due to the rotated visual–feedback, and they are estimated from the angular error of the return movement (angle α in **Figure 1B**). When the sensed position deviated in the CCW or CW direction from the actual position of the hand, the angular deviation (α’) had a positive or negative sign, respectively. Individual means and standard deviations of the angular deviations were computed for each delay condition and each visual–feedback rotation across trials including both judged directions (cursor and hand) because the 3rd strokes were made before the direction to be judged was instructed. The angular deviations α’ were subjected to the same linear regressions as the angular deviations of the judgments of hand direction from the actual direction. The slope parameters of these regressions served as implicit measures of the bias of sensed hand direction toward the direction of the cursor.

The data were screened for outliers both among trials and among participants. Based on the linear regressions, trials with angular deviations outside the range of predicted deviations ± 3 standard deviations of the residuals were eliminated as outliers. As a result of the screening of the explicit measures of hand and cursor direction, 0.20% of all trials were removed from all analyses. The screening of the implicit measure of hand direction resulted in a removal of 0.20% of trials. Subsequently, the bias parameters for each type of measure (explicit cursor, explicit hand, implicit hand), each delay condition, and each participant were screened for outliers. Means and standard deviations across all participants were calculated for the three types of measure and the two delay conditions, and bias parameters outside the range of mean ± 3 standard deviations were defined as outliers. These computations were repeated until no further outliers were found. As the result, three participants were identified as having outliers for the explicit measure of cursor direction and were excluded from all analyses.

The bias parameters, as estimated by the slopes of the linear regressions of angular deviations on visual–feedback rotation, neglected the pointing errors of the 2nd strokes to the remembered targets (T2). To exclude a possible role of such errors for our results, we estimated the biases also in a second way, using the estimated angular deviations for a pointing error of zero at each visual–feedback rotation instead of the directly observed angular deviations. The results obtained with these estimates of bias parameters confirmed the findings with the more direct estimates. Both the computation of these estimates and the results obtained with them are reported in detail as Supplementary Material.

For statistical analysis, individual regression coefficients (our explicit and implicit measures of biases of sensed hand and cursor directions) were subjected to a 2 (delay condition: immediate vs. delayed) × 2 (type of measure: hand-explicit vs. hand-implicit) repeated-measures ANOVA. Individual standard deviations were subjected to a 2 (delay condition: immediate vs. delayed) × 2 (type of measure: hand-explicit vs. hand-implicit) × 6 (visual–feedback rotation) repeated-measures ANOVA. The explicit measure of cursor direction was analyzed separately, with a *t*-test for the regression coefficients and a 2 (delay condition: immediate vs. delayed) × 6 (visual–feedback rotation) repeated-measures ANOVA for the individual standard deviations. When appropriate, *post hoc* comparisons were performed using *t*-tests with Bonferroni correction (α = 0.05). Mauchly’s test was used to determine if sphericity was violated and was found non-significant for all relevant ANOVA results.

### Results

Our main interest is in the explicitly and implicitly assessed biases of hand direction toward the direction of the cursor. Especially, we are interested in their changes induced by the delay and the associated variation of the relative reliabilities of visual information on cursor position and proprioceptive information on hand position. In addition, we report the intra-individual variability of implicit and explicit measures of hand direction and the findings for the explicit measure of cursor direction.

#### Explicit and Implicit Measures of the Bias of Sensed Hand Direction

**FIGURE 2:**
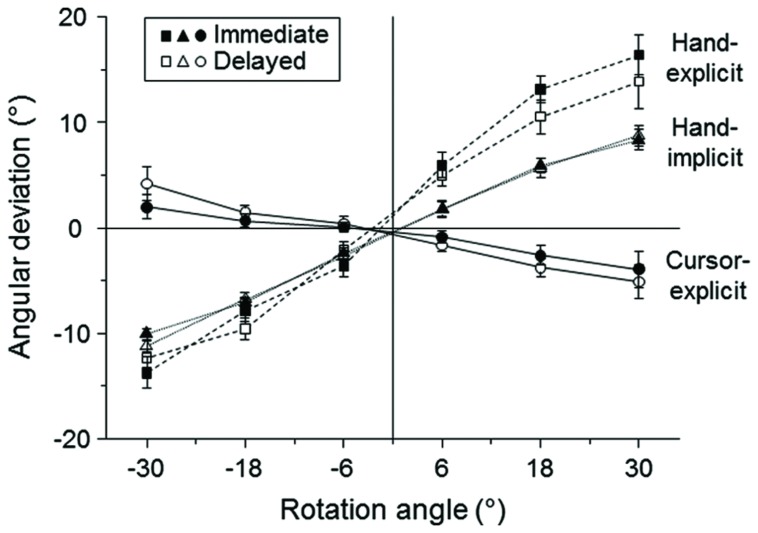
**Mean explicit and implicit measures as a function of the rotation of visual–feedback.** The mean values across all participants are plotted for explicit measures of cursor direction (circles) and hand direction (squares) and implicit measures of hand direction (triangles). Open and filled symbols refer to the delayed and immediate conditions, respectively. The error bars represent the SE.

The mean angular deviation of the judged hand direction from the physical direction showed steep positive slopes as a function of the visual–feedback rotation (**Figure 2**, squares). This indicates a strong explicitly assessed bias toward the direction of the cursor. Turning to the implicit measure of the bias of hand direction, the mean angular deviation between the directions of the 2nd and 3rd stroke had positive slopes as a function of the visual-feedback rotation (**Figure 2**, triangles), indicating a bias toward the cursor direction. These slopes were less steep than those observed for the explicit measure (**Figure 2**, squares).

The means (SE) of the individually estimated biases (slopes of the linear regressions) for the explicit measure of hand direction toward the direction of the cursor were 0.531 (0.043) for the immediate and 0.440 (0.053) for the delayed condition. For the implicitly assessed biases of hand direction, the means were 0.306 (0.015) and 0.325 (0.015) for the immediate and delayed condition, respectively. A 2 (delay condition: immediate vs. delayed) × 2 (type of measure: hand-explicit vs. hand-implicit) ANOVA revealed a significant main effect of type of measure [*F*(1,24) = 17.6, *P* < 0.001]. The main effect of delay was not significant [*F*(1,24) = 2.6, *P >* 0.05]. Most importantly, the significant interaction of delay condition and type of measure [*F*(1,24) = 6.4, *P <* 0.05] indicates different effects of the delay on the explicitly and implicitly assessed biases. Whereas the explicitly assessed bias was stronger in the immediate than in the delayed condition (**Figure 2**, squares), the implicitly assessed bias was not (**Figure 2**, triangles). A *post hoc* analysis with Bonferroni correction revealed that the difference between the immediate and the delayed condition was almost significant for the explicit measure (*P* = 0.08), but not for the implicit one (*P* = 0.25).

#### Intra-individual Variability of Biases of Sensed Hand Direction

**FIGURE 3:**
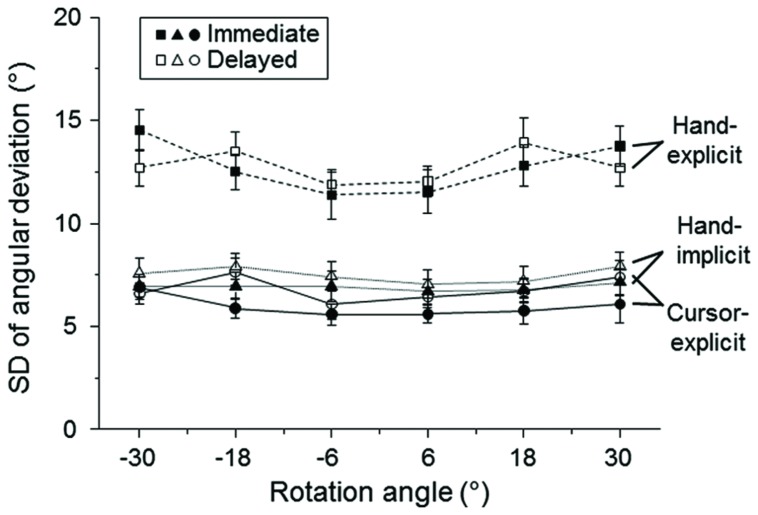
**Mean standard deviations (SD) of the explicit and implicit measures as a function of the rotation of visual–feedback.** The mean SDs across participants are plotted for explicit measures of cursor direction (circles) and hand direction (squares) and implicit measures of hand direction (triangles). Open and filled symbols refer to the delayed and immediate conditions, respectively. The error bars represent the SE.

The mean intra-individual standard deviations (**Figure 3**) were subjected to a 2 (delay condition: immediate vs. delayed) × 2 (type of measure: hand-explicit vs. hand-implicit) × 6 (visual–feedback rotation) ANOVA. There were significant main effects of type of measure [*F*(1,24) = 97.7, *P* < 0.001] and of visual–feedback rotation [*F*(5,120) = 4.5, *P* < 0.01], but the delay effect [*F*(1,24) = 0.5, *P* > 0.05] and interactions involving this factor were all non-significant. Thus, intra-individual variability was independent of delay. The individual standard deviations were small for the implicit measure of hand direction (**Figure 3**, triangles), but those for the explicit measure were much larger (**Figure 3**, squares).

#### Explicit Measure of Sensed Cursor Direction

The slope of the mean angular deviation of the judged cursor direction from the physical direction as a function of the visual-feedback rotation was negative and small in absolute terms (**Figure 2**, circles). This indicates a weak bias toward the direction of the hand. The mean (SE) biases of cursor judgments toward the direction of the hand were negative for both the immediate condition (-0.059 ± 0.013) and the delayed condition (-0.099 ± 0.015), and they were significantly different from each other [*t*(24) = 3.0, *P* < 0.01]. The delay effect (calculated as difference between the delayed and immediate conditions) of -0.040 (SE: 0.013) found for the explicit measure of cursor direction was not significantly different from the delay effect of -0.091 (0.042) found for the explicit measure of hand direction [*t*(24) = 1.2, *P* > 0.05]. Thus, whereas the (positive) bias of judgments of hand direction toward the direction of the cursor became weaker after the delay, the (negative) bias of cursor judgments toward the direction of the hand became stronger by an equivalent amount.

The mean intra-individual standard deviations of the explicit measure of cursor direction were small, as shown in **Figure 3** (circles). An ANOVA with the factors delay condition (immediate vs. delayed) and visual–feedback rotation revealed only a significant main effect of delay [*F*(1,24) = 6.7, *P <* 0.05]. The intra-individual variability was larger in the delayed than in the immediate condition.

### Discussion

The variation of the relative reliabilities of visual information on cursor direction and proprioceptive information on hand direction had different effects on explicit and implicit measures of the bias of sensed hand direction at the end of an aiming movement toward the direction of the cursor. Relative reliabilities were varied by means of a 6-s period at the end of aiming, during which proprioceptive information was available, but visual information was not. As a consequence, the explicitly measured bias of sensed hand direction toward the direction of the cursor was reduced, whereas – in a complementary manner – the explicitly measured bias of sensed cursor direction toward the direction of the hand became stronger. In contrast, the implicit measure of the bias of sensed hand direction toward the direction of the cursor remained unchanged. Thus, our manipulation of the relative reliabilities of proprioceptive and visual information succeeded in producing different effects on our explicit and implicit measures of hand direction.

The different effects of the variation of the relative reliabilities on explicit and implicit measures suggest different principles of sensory coupling. Perhaps the most basic principle of sensory integration is minimization of the variance of a weighted average of sensory signals, with the weights being adjusted to the respective reliabilities of the individual signals (cf. Ernst, 2006, 2012). This principle is likely to be supplemented with other influences (cf. Sober and Sabes, 2005). In a cursor-control task, for example, visual information on cursor position is critical for determining the success of an action because the target is typically given by vision. From the perspective of task performance, which is generally subject to conscious awareness, the position of the cursor matters, whereas the position of the hand is irrelevant. However, the position of the hand is likely to matter for movement control, at least when movements are coded in terms of directions and amplitudes (e.g., Gordon et al., 1994; Rossetti et al., 1995; Vindras and Viviani, 1998, 2002; Wang and Sainburg, 2005). Because of the different roles of visual and proprioceptive information in a cursor-control task, and in tool use more generally, principles of sensory coupling might differ between measures that rely on conscious awareness (explicit measures) or are derived from motor behavior (implicit measures), respectively.

Turning to the specific differences observed between explicit and implicit measures of hand direction, our findings are consistent with the hypothesis that the measures differ with respect to the times at which sensory coupling occurs. Implicit measures should reflect rather continuous sensory coupling throughout the duration of the aiming movement, which would leave only a weak influence of the final unimodal proprioceptive information on hand position in the delayed condition. Explicit measures, in contrast, should reflect sensory coupling just before a judgment is provided and thereby be based on the relative reliabilities of (memorized) sensory information at a certain time after the end of the movement, where the relative reliabilities of proprioceptive and visual information clearly differed between the immediate and the delayed condition. A possible alternative to this hypothesis is that the weighting of different sensory modalities tapped by the implicit measure is fundamentally insensitive to the relative reliabilities of the coupled signals. However, this alternative appears unlikely, given the broad evidence of reliability-based weighting not only for psychophysical judgments (e.g., Fetsch et al., 2012).

Consistent with previous findings (Rand and Heuer, 2013), here we found again (1) considerably stronger explicitly measured biases of hand direction than implicitly measured biases and (2) a much stronger intra-individual variability of explicitly measured biases than of implicitly measured ones. These findings, together with the new observation of different effects of the relative reliabilities on explicit and implicit measures, are consistent with the hypothesis of distinct representations of hand position involved in conscious awareness, as tapped by our explicit measure, and movement control, as tapped by our implicit measure.

## Experiment 2

In the second experiment, we examine the effects of the sequence of trials with different judgments (cursor or hand) on explicit and implicit measures of hand direction. Trial history is known to affect various behaviors, such as grip force production (Johansson and Westling, 1988; Lukos et al., 2013), reaction time (Kirby, 1980; Song and Nakayama, 2007), aiming movements (Zelaznik et al., 1983; Khan et al., 2002; Song and Nakayama, 2007; Cheng et al., 2008), reach-to-grasp movements (Rand et al., 2004; Whitwell et al., 2008; Whitwell and Goodale, 2009), and saccadic eye movements toward visual targets (Fecteau and Munoz, 2003). Implicitly and explicitly measured biases of hand direction are no exception: Rand and Heuer (2013) found that they were affected by the immediate trial history, that is, by the local context, in opposite directions. The present experiment was designed to explore the effects of global rather than local context on implicit and explicit measures of hand direction.

The exploration of the effects of global context was motivated by the following considerations. First, a more or less continuously updated representation of hand position is needed for movement control, and it should be tapped by our implicit measure. This representation and its updating likely involve rather autonomous processes that are cognitively impenetrable (cf. Whitwell et al., 2008) and probably insensitive to the global context, as is the case with other motor behaviors such as the grasping component controlled implicitly during reach-to-grasp movements (Whitwell et al., 2008; Whitwell and Goodale, 2009). Explicit measures, on the other hand, involve psychophysical judgments which are well known to be affected by context (cf. Poulton, 1989). Thus, this consideration suggests the hypothesis that global context should affect explicit, but not implicit measures. Second, (conscious) expectations of a judgment of hand or cursor direction could serve to direct attention to proprioceptive or visual information, respectively, and thereby to modulate the asymmetry of the biases. Such an effect of predictability could be restricted to explicit measures of bias. According to this consideration, predictability of the forthcoming judgments should affect explicitly assessed biases, but not implicitly assessed ones. Third, implicit measures, but not explicit ones, could be affected by the preceding judgments (cf. Rand and Heuer, 2013). Thus, when global contexts differ with respect to preceding judgments only, implicitly assessed biases, but not explicitly assessed biases, should be modulated.

We studied the effects of global context by means of a methodology used by Zelaznik et al. (1983) and others (Khan et al., 2002; Song and Nakayama, 2007; Whitwell et al., 2008). The principle is to compare two types of trials under three different conditions with blocked, alternated, and randomized order. In the present experiment, the two types of trials are those with judgments of hand and cursor direction. With blocked trials, the global context is homogeneous. Each trial is preceded by a number of trials with the same judgment, and prediction of the judgment required in the next trial is straightforward. With alternating trials, the global context is heterogeneous, though predictable. The heterogeneous global context could affect information processing in various ways (Los, 1996). One group of participants underwent these conditions in which the sequence of judgments was predictable. A second group of participants underwent a randomized order of judgments. Here the global context is not only heterogeneous, but the judgment required in the next trial is unpredictable in addition.

### Materials and Methods

#### Participants

Fifty healthy right-handed participants signed informed consent. They were randomly assigned to Group 1 (*n* = 25, mean ± SD: 24.8 ± 3.6 years; 10 males and 15 females) or Group 2 (*n* = 25, mean ± SD: 24.5 ± 3.2 years; 10 males and 15 females). The study was conducted in accordance with the Declaration of Helsinki and with the general approval by the ethics committee of the Leibniz Research Centre for Working Environment and Human Factors.

#### Apparatus and Procedure

The experimental apparatus was the same as in Experiment 1, and the procedure during each trial was the same as in the immediate condition of Experiment 1 with two exceptions. First, the range of the rotation angles was reduced in Experiment 2 because judgment variability was somewhat increased at the largest rotation angles (-30° and 30°) of Experiment 1 (cf. **Figure 3**). The six angles of rotated visual–feedback for the 2nd strokes ranged between -25° and +25° (CW direction: -25°, -15°, -5°; CCW direction: 5°, 15°, 25°). Second, unlike Experiment 1, no beep sound was provided to signal that participants should start the 3rd stroke.

The global context was varied across two groups. In Group 1, the sequence of judgments of hand and cursor was predictable. There were three different conditions. In the blocked-cursor condition (72 trials), cursor direction had to be judged in all trials; in the blocked-hand condition (72 trials), hand direction had to be judged in all trials; in the alternated condition, judgments of cursor and hand direction alternated (72 trials for each type of judgment, totaling 144 trials). Participants of Group 1 were informed at the beginning of each condition that all trials required the same judgment for the blocked-cursor and blocked-hand conditions, and that the hand and cursor judgments alternated across trials in the alternated condition. In Group 2, there was only a single condition (randomized) in which judgments of cursor and hand direction were mixed as in the alternated condition, but their sequence across trials was random and thus unpredictable (144 trials for each judgment, totaling 288 trials).

Before data recording, there were eight familiarization trials that included the procedure without the visual–feedback rotation and/or without the judgment and four practice trials that included all the procedure (two trials for each type of judgment). An experimental block included 1 warm-up trial and 36 experimental trials (six trials for each of the six angular rotations). Each of the blocked-cursor and blocked-hand conditions included two blocks, and the alternated condition included four blocks. The order of the three conditions was randomized across participants of Group 1. The randomized condition of Group 2 included eight blocks of trials. A total of 288 experimental trials were recorded and analyzed for each participant. A few minutes of break were inserted after each block.

#### Data Analysis

Data analysis was basically identical to that of Experiment 1 with one exception. Namely, individual means and standard deviations of the angular deviation of the direction of the 3rd stroke from the direction of the 2nd stroke were computed not only for each context condition and each visual–feedback rotation, but also for each judged direction (cursor or hand). This is because in Experiment 2, the 3rd strokes were made not always before the direction to be judged was instructed. The data were screened for outliers both among trials and among participants as in Experiment 1. As a result of the screening of the explicit measures, 0.49% of trials were removed from all analyses. The screening of the implicit measure resulted in a removal of 0.32% of trials. Subsequently, the individual bias parameters were screened for outliers based on means and standard deviations across all participants for each type of measure (explicit cursor, explicit hand, and implicit hand) and each context condition. Two participants of each group were identified as having outliers for the explicit measure of cursor bias. These participants were excluded from all analyses.

For the statistical analysis, individual regression coefficients (as measures of explicit and implicit biases) and standard deviations were used as in Experiment 1. In a first step, we analyzed explicit and implicit measures of hand direction. When comparing the blocked and alternated conditions of Group 1, mainly ANOVAs with different within-participant factors (such as visuo-motor rotation, type of measure, and context condition) were used. When comparing the alternated and randomized conditions, ANOVAs with a between-participant factor (context condition) and different within-participant factors (such as visuo-motor rotation and type of measure) were used. In a second step, explicit measures of cursor direction were analyzed. The blocked and alternated conditions of Group 1 were compared by using a paired *t*-test for the regression coefficients and an ANOVA with within-participant factors (context condition and visuo-motor rotation) for the individual standard deviations. The alternated and randomized conditions were compared by using a *t*-test for the regression coefficients and an ANOVA with a between-participant factor (context condition) and a within-participant factor (visuo-motor rotation) for the individual standard deviations. Mauchly’s test was used to determine if sphericity was violated and was found non-significant for all relevant significant ANOVA results.

### Results

The main purpose of Experiment 2 was to test whether the explicit and implicit measures of the bias of hand direction toward the direction of the cursor are affected differently by global context. Therefore, we report first the differences of these measures between the blocked and alternated predictable conditions and between the alternated (predictable) and randomized (unpredictable) conditions. Next, we report the intra-individual variability of implicit and explicit measures of hand direction and finally the findings on the explicit measure of cursor direction.

#### Explicit and Implicit Measures of the Bias of Sensed Hand Direction

**FIGURE 4:**
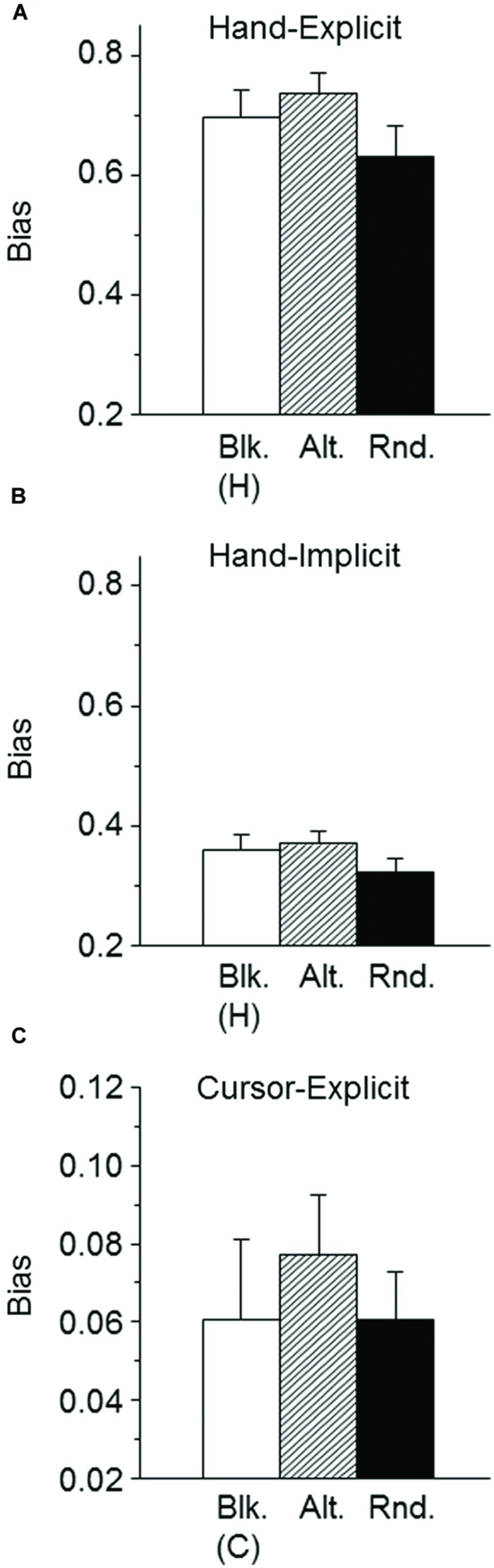
**Mean explicitly measured biases of hand direction **(A)**, implicitly measured biases of hand direction **(B)**, and explicitly measured biases of cursor direction **(C)**.** The mean values across participants are plotted for the blocked-hand [Blk. (H)], blocked-cursor [Blk. (C)], and alternated (Alt.) conditions of Group 1 as well as the randomized (Rnd.) condition of Group 2. The error bars represent the SE.

First, the effects of global context on implicit and explicit measures of hand direction were examined by comparing the blocked-hand and alternated conditions of Group 1. Similar to Experiment 1 (**Figure 2**), there was a generally stronger explicit-measure bias than implicit-measure bias toward the direction of the cursor. The means of the individually estimated biases across two context conditions were 0.716 for the explicit measure (**Figure 4A**) and 0.365 for the implicit measure (**Figure 4B**). A 2 (context: blocked-hand vs. alternated) × 2 (type of measure: hand-implicit vs. hand-explicit) ANOVA revealed a significant main effect of type of measure [*F*(1,22) = 69.5, *P* < 0.001]. Although both the explicit and the implicit measure of the bias were slightly larger in the alternated than in the blocked condition (**Figures 4A,B**), the main effect of context condition was not significant [*F*(1,22) = 2.5, *P* > 0.05]. The interaction of context condition and type of measure was not significant as well [*F*(1,22) = 0.8, *P* > 0.05]. Thus, there was no evidence of a different effect of global context on explicit and implicit measures of the bias of sensed hand direction toward the direction of the cursor.

Second, the effects of global context on implicit and explicit measures of hand direction were examined by comparing the alternated condition of Group 1 (predictable) and the randomized condition of Group 2 (unpredictable). A 2 (context: alternated vs. randomized) × 2 (type of measure: hand-implicit vs. hand-explicit) ANOVA revealed significant main effects both of type of measure [*F*(1,44) = 115.5 *P* < 0.001] and context [*F*(1,44) = 4.3, *P* < 0.05]. The interaction was not significant [*F*(1,44) = 0.8, *P* > 0.05]. Both for the implicit and the explicit measure, the bias of sensed hand direction toward the cursor direction was less in the randomized condition than in the alternated condition (**Figures 4A,B**), and there was no evidence of different modulations of explicit and implicit biases by the global context.

#### Intra-individual Variability of Biases of Sensed Hand Direction

**FIGURE 5:**
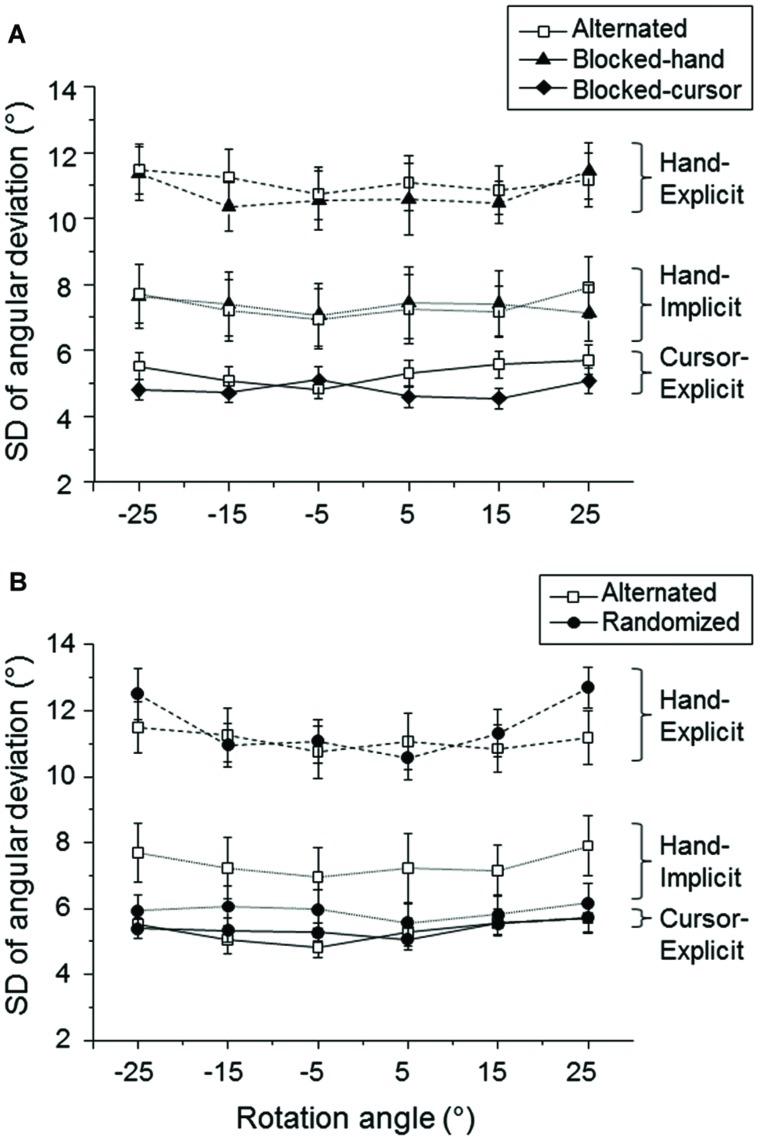
**Mean standard deviations (SD) of the explicit and implicit measures as a function of the rotation of visual–feedback.** **(A)**. Comparisons between blocked and alternated conditions of Group 1. The values are plotted for the blocked-hand (filled triangles), blocked-cursor (filled diamonds), and the alternated (open squares) conditions. **(B)**. Comparisons between the alternated condition of Group 1 (open squares) and the randomized condition of Group 2 (filled circles). The error bars represent the SE.

First, we compared the blocked condition with the alternated condition of Group 1. The mean intra-individual standard deviations were subjected to a 2 (context: blocked-hand vs. alternated) × 2 (type of measure: hand-explicit vs. hand-implicit) × 6 (visual–feedback rotation) ANOVA. Only the main effect of type of measure was significant [*F*(1,22) = 16.2, *P* < 0.01]. Similar to the results of Experiment 1, the individual standard deviations of the implicit measure (**Figure 5A**, hand-implicit) were substantially smaller than those of the explicit measure (**Figure 5A**, hand-explicit). The global context did not affect the variability.

Second, we compared the alternated condition of Group 1 with the randomized condition of Group 2 (**Figure 5B**, hand-explicit, hand-implicit). The mean intra-individual standard deviations were subjected to a 2 (context: alternated vs. randomized) × 2 (type of measure: hand-explicit vs. hand-implicit) × 6 (visual–feedback rotation) ANOVA. The main effect of type of measure was significant [*F*(1,44) = 67.2, *P* < 0.001], and so was the main effect of visual–feedback rotation [*F*(5,220) = 4.9, *P* < 0.01]. All interaction effects were not significant, giving no evidence of different context effects on explicit and implicit measures of bias.

#### Explicit Measure of Sensed Cursor Direction

Similar to the results of Experiment 1 (**Figure 2**, cursor-explicit), the mean angular deviations of the judged cursor directions from the corresponding physical directions showed slightly negative slopes as a function of the visual-feedback rotation. This indicates a weak bias of the judgments of cursor direction toward the direction of the hand. The mean individual biases were multiplied with -1 to obtain positive values which are plotted in **Figure 4C.** Regarding the effects of global context, the explicit measures of the bias of sensed cursor direction toward the direction of the hand did not differ between the alternated and the blocked-cursor conditions [*t*(22) = 1.09, *P* > 0.05]. Similarly, the biases did not differ between the alternated and randomized conditions [*t*(44) = 0.86, *P* > 0.05]. Nevertheless, it is noteworthy that the variations of the biases of cursor judgments across context conditions (**Figure 4C**) were similar to the variations of the biases of hand judgments (**Figure 4A**). Unlike in Experiment 1, there was no indication that a stronger bias of hand judgments in one of the experimental conditions was accompanied by a weaker bias of cursor judgments.

The mean intra-individual standard deviations of the explicit measure of the bias of sensed cursor direction were generally small (**Figure 5A,B**, cursor-explicit). Regarding the effects of global context, a 2 (context: blocked-cursor vs. alternated) × 6 (visual–feedback rotation) ANOVA revealed only a significant main effect of context [*F*(1,22) = 4.5, *P <* 0.05]. The variability of cursor judgment was larger in the alternated condition than in the blocked-cursor condition (**Figure 5A**). A 2 (context: alternated vs. randomized) × 6 (visual–feedback rotation) ANOVA revealed only a significant main effect of feedback rotation [*F*(5,220) = 2.5, *P <* 0.05]. Thus, the variability of cursor judgments was quite similar in the alternated and randomized conditions (**Figure 5B**).

### Discussion

Experiment 2 was designed to explore contrasting effects of global context on explicit and implicit judgments of hand direction. Such contrasting effects turned out to be essentially absent. Instead, the global context effects were quite similar for explicit and implicit measures of sensed hand direction. We shall discuss the absence of contrasting effects and the observed common effects in turn.

The three different global contexts in terms of trials with judgments of hand and cursor direction differed with respect to the heterogeneity of the judgments in a block of trials and with respect to their predictability. According to our initial considerations, both these factors could have affected the explicit and implicit judgments differently. For example, (conscious) expectation of a judgment of hand direction could have served to direct attention to proprioceptive information, increasing the weight of this signal in sensory coupling so that the bias of sensed hand direction toward the direction of the cursor would be reduced. Such an effect could be limited to explicit measures. The absence of such an effect is in line with the claim that the weights of sensory coupling are essentially independent of attending to the one or the other signal (cf. Helbig and Ernst, 2008). Similarly, a judgment of hand direction in a preceding trial could have facilitated processing of proprioceptive information, again increasing its weight in sensory coupling. Such an effect could have been limited to the implicit measure. Again there was no indication of such a differential modulation of biases. Finally, the implicit measure, but not the explicit one, could have been insensitive to global context in principle. The present findings also do not substantiate this tentative hypothesis. Taken together, they also cast doubt on the robustness and/or generality of the different sequential effects for the explicit and implicit measures of hand direction found by Rand and Heuer (2013).

The effects of global context were not only similar for explicit and implicit measures of hand direction, but also for explicit measures of hand and cursor direction, even though for the explicit measure of cursor direction, the variations across context conditions were small and not statistically significant. This pattern of results is different from Experiment 1 where the delay produced opposite effects on the explicit measures: it reduced the bias of sensed hand direction toward the direction of the cursor and increased the bias of sensed cursor direction toward the direction of the hand. Thus, the delay of Experiment 1 affected primarily the asymmetry of the bias, whereas the global contexts of Experiment 2 affected primarily the strength of coupling (sum of the biases of sensed hand and cursor directions). In formal models of sensory coupling (e.g., Bresciani et al., 2006), coupling strength has been claimed to depend on a coupling prior, which reflects the probability of knowing the relation between the different sensory signals (Ernst, 2006).

Coupling strength varied systematically across the context conditions of Experiment 2. Most conspicuously, it was stronger in the alternated condition than in the randomized condition. Thus, in the randomized condition, participants were more proficient in keeping visual information on cursor position and proprioceptive information on hand position separated. We suggest that this is a consequence of higher effort or investment of more cognitive resources. Randomness of the sequence of judgments of cursor and hand directions could enhance the subjective difficulty of the task and thereby increase motivation. The relation between task difficulty and effort or motivation has a long history in Psychology (see Brehm and Self, 1989, for review). This relation has even been invoked to account for apparently paradoxical increases of performance when the task became harder to perform (e.g., Düker, 1963). Findings such as these imply that increased effort goes along with the allocation of additional cognitive resources (cf. Kahneman, 1973). Although we are not aware of direct evidence related to effort and sensory coupling, there is some indirect evidence on the availability of cognitive resources, as varied by age of the participants, and sensory coupling. At older adult age, when cognitive resources can be conceived as being reduced in comparison with young adult age (e.g., Salthouse, 1988), we found stronger biases of judged directions in a cursor-control task (Rand and Heuer, 2013) and poorer discriminability of hand and cursor directions (Rand et al., 2013). These findings are consistent with the hypothesis that reduced cognitive resources result in a reduced proficiency to keep visual information on cursor position and proprioceptive information on hand position separated.

## General Discussion

The present findings add to the understanding of sensory coupling in cursor-control tasks and, more generally, in tool-use tasks in which visual and proprioceptive information refer to different objects (the hand and the effective part of a tool such as the cursor in a cursor-control task). Across different dependent measures such as (intentional) reproductions of movement distances or psychophysical judgments of cursor and hand positions as well as for positions, amplitudes and directions, the general finding is an assimilation, which is typically asymmetric (Ladwig et al., 2013; Rand and Heuer, 2013; Kirsch et al., 2015). Although it is not always clear that tool use, in particular the presence of a kinematic transformation, is critical for the assimilation, it is worth noting that assimilation of concurrent visual and proprioceptive signals is not a universal phenomenon. For example, when an independently moving visual stimulus (cursor) was concurrently presented with aiming actions, a contrast effect has been observed where the seen cursor direction was shifted away from the felt hand direction (Zwickel et al., 2007, 2008, 2010a,b). This contrast effect was flipped to assimilation under specific circumstances (Zwickel et al., 2010a). Even though the contrast effect was specific to independent visual and proprioceptive inputs, such a flipping reveals the complex nature of sensory coupling associated with manual actions and the need of a careful examination of factors that affect the cross-modal influences.

One such factor is the measure by which cross-modal influences are assessed. The type of measure should play a role as soon as sensory coupling does not only depend on the reliabilities of the coupled signals and the *a priori* knowledge of the relation between these signals, but also on the purpose or use of the combined signal (cf. Knill, 2005; Sober and Sabes, 2005; Greenwald and Knill, 2009). Here we draw a distinction between an explicit measure, which is based on psychophysical judgments and implies subjective awareness of the judged positions of hand and cursor, and an implicit measure, which is derived from motor behavior and does not imply subjective awareness of hand position. We found that the two types of measure are differently sensitive to variation of the relative reliabilities of proprioceptive and visual information, in particular, to a boost of the reliability of proprioceptive information on the end position of a movement by unimodal presentation. In contrast, they turned out to be similarly affected by the variation of coupling strength induced by global context, in particular, the sequences of psychophysical judgments of hand and cursor directions.

According to the current findings, our explicit and implicit measures differ with respect to the reliability-based weighting of proprioceptive and visual information. The different weights could be a consequence of different reliabilities of the coupled individual signals at different times during a movement. The implicit measure is likely based on a continuously updated representation of hand position, and the reliability of hand-position information could be enhanced during the movement by efferent contributions (cf. Paillard and Brouchon, 1968; Craske and Crawshaw, 1975; Wann and Ibrahim, 1992). With such a continuously updated representation, the boost of the reliability of proprioceptive information after the end of the movement, which we implemented in the delayed condition of Experiment 1, should have only little or no effect. The explicit measure, in contrast, is likely based on the sensory information available at the time the psychophysical judgment is required. Thus, reliabilities during the movements should be of little importance, but reliabilities just before the judgments – and after the end of the movement – should be.

The current findings suggest that our explicit and implicit measures of sensed hand direction share variations of coupling strength, which should be related to prior knowledge of the relation between hand and cursor movements (cf. Ernst, 2012). This so-called coupling prior seems to be affected by the effort invested in task performance, with a higher effort going along with a weaker coupling. A weaker coupling implies less *a priori* redundancy of visual and proprioceptive information, so that there is a higher demand on the processing of different concurrent sensory signals. In normal everyday life, there is a tight relation between the directions of hand movements and cursor motions. Thus, a high coupling strength would be appropriate. In the experimental task, however, the relation between the directions of hand movements and cursor motions is less tight because of the random visuo-motor rotations. Therefore, a weaker coupling strength would be appropriate. Appropriate adjustments of the coupling prior could also be facilitated by higher effort or increased cognitive resources.

## Author Contributions

Conceived and designed the experiments: MR and HH. Performed the experiments: MR. Analyzed the data: MR and HH. Wrote the paper: MR and HH.

## Conflict of Interest Statement

The authors declare that the research was conducted in the absence of any commercial or financial relationships that could be construed as a potential conflict of interest.
