# Supplementary material for: Effects of Reliability and Global Context on Explicit and Implicit Measures of Sensed Hand Position in Cursor-Control Tasks
Source: Front Psychol. 2016 Jan 12;6:2056. doi: 10.3389/fpsyg.2015.02056 (PMC4709824; doi:10.3389/fpsyg.2015.02056)
Supplement: Supplementary file 1 [file Data_Sheet_1.PDF]

Supplementary Material

Effects of reliability and global context on explicit and implicit measures of sensed hand position in cursor-control tasks

Miya K. Rand*, Herbert Heuer
*** Correspondence:**
Miya K. Rand:
rand@ifado.de

1 Additional analyses: pointing errors of the 2nd stroke and estimated bias parameters for zero pointing errors.

There was a possibility that the pointing errors of the 2nd strokes to the remembered targets (T2) might have some unexpected influence on the implicit and explicit measures of sensed hand directions. Thus, we first checked the accuracy of the 2nd strokes as a function of the visual-feedback rotation. We found linear relations with pointing errors (i.e., difference between the T2 and the end of the 2nd stroke) of about -1.4° at a visual-feedback rotation of -30° and about +1.7° at a visual-feedback rotation of +30° in Experiment 1. There was no difference between the immediate and the delayed condition. A 2 (delay condition: immediate vs delayed) x 6 (visual-feedback rotation) ANOVA revealed a significant main effect of visual-feedback rotation ($F(5,120)=16.0$, $P<0.001$ based on uncorrected d.f., Greenhouse-Geisser $\varepsilon=0.405$), but the main effect of delay condition ($F(1,24)=0.2$, $P>0.05$) and the interaction ($F(5,120)=0.9$, $P>0.05$, $\varepsilon=0.638$) were not significant. In Experiment 2, we found linear relations of pointing errors to visual-feedback rotation with pointing errors of about -2.5° at a visual-feedback rotation of -25° and about +2.0° at a visual-feedback rotation of +25° across the blocked and alternated conditions. A 2 (judged direction: cursor vs hand) x 2 (context: blocked vs alternated) x 6 (visual-feedback rotation) ANOVA revealed a significant main effect of visual-feedback rotation ($F(5,110)=18.0$, $P<0.001$, $\varepsilon=0.240$) and a significant interaction between context and feedback rotation ($F(5,110)=4.1$, $P<0.01$, $\varepsilon=0.604$). The linear relation was stronger for the blocked condition compared to the alternated condition. The context main effect and all other interactions were not significant. For the alternated and randomized conditions, the pointing errors were about -1.8° at a visual-feedback rotation of -25° and about +1.8° at a visual-feedback rotation of +25° across the two context conditions. A 2 (judged direction: cursor vs hand) x 2 (context: alternated vs randomized) x 6 (visual-feedback rotation) ANOVA revealed only a significant main effect of visual-feedback rotation ($F(5,220)=31.1$, $P<0.001$, $\varepsilon=0.288$). In summary, the pointing error was linearly altered depending on visual-feedback rotation angles in both experiments. This feature did not differ between the delay conditions (Exp. 1) or between the global contexts of alternated and randomized conditions (Exp. 2). The linear dependence of the pointing error on the rotation angles was strengthened in the global context of blocked trials (Exp. 2).

In order to estimate the biases of sensed hand position towards the position of the cursor and of sensed cursor position towards the position of the hand, we statistically removed the different pointing errors at the different visual-feedback rotations. For each participant, each type of measure (hand implicit, hand explicit, cursor explicit), each delay condition in Exp. 1 or context condition in Exp. 2, and each visual-feedback rotation, we computed the linear regression of measured angular deviation (direction of 3rd stroke from that of 2nd stroke, judged hand direction from physical

direction, judged cursor direction from physical direction) on pointing errors. For each visual-feedback rotation, the intercept of the regression of angular deviation on pointing error is an estimate of the angular deviation at a pointing error of zero. We used these intercepts to replace the directly observed angular deviations in the regressions on the visual-feedback rotations. Different from the biases reported in the main article, the slopes of these regressions estimate the biases unaffected by different pointing errors at different visual-feedback rotations. We reran the statistical analyses with these bias estimates, as detailed below. There were no substantial deviations from the analyses reported in the main article.

1.1 Results Experiment 1

The means (SE) of the individually estimated biases (slopes of the linear regressions) for the explicit measure of hand direction toward the direction of the cursor were 0.512 (0.051) for the immediate and 0.395 (0.059) for the delayed condition. For the implicitly assessed biases of hand direction, the means were 0.325 (0.016) and 0.347 (0.016) for the immediate and delayed condition, respectively. A 2 (delay condition: immediate vs delayed) x 2 (type of measure: hand-explicit vs hand-implicit) ANOVA revealed a significant main effect of type of measure ($F(1,24)=5.9$, $P<0.05$). Most importantly, the interaction between delay condition and type of measure ($F(1,24)=9.8$, $P<0.01$) was significant, and so was the main effect of delay ($F(1,24)=4.7$, $P<0.05$). A post-hoc analysis with Bonferroni correction revealed that the difference between the immediate and the delayed condition was significant for the explicit measure ($P<0.05$), but not for the implicit measure ($P>0.05$).

The mean (SE) biases of cursor judgments toward the direction of the hand were negative for both the immediate condition (-0.059±0.013) and the delayed condition (-0.124±0.018), and they were significantly different from each other ($t(24)=4.3$, $P<0.001$). The delay effect (calculated as difference between the delayed and immediate conditions) of -0.065 (SE: 0.015) found for the explicit measure of cursor direction was not significantly different from the delay effect of -0.117 (0.042) found for the explicit measure of hand direction ($t(24)=1.4$, $P>0.05$).

1.2 Results Experiment 2

The means (SE) of the individually estimated biases (slopes of the linear regressions) for the explicit measure of hand direction toward the direction of the cursor were 0.679 (0.047) for the blocked and 0.704 (0.033) for the alternated condition. For the implicitly assessed biases of hand direction, the means were 0.392 (0.025) and 0.390 (0.022) for the blocked and alternated condition, respectively. A 2 (context: blocked-hand vs alternated) x 2 (type of measure: hand-implicit vs hand-explicit) ANOVA revealed a significant main effect of type of measure ($F(1,22)=45.1$, $P<0.001$). The main effect of context condition ($F(1,22)=0.4$, $P>0.05$) and the interaction of context condition and type of measure ($F(1,22)=0.5$, $P>0.05$) were not significant. For the randomized condition of Group 2 (unpredictable), the means (SE) of the biases were 0.581 (0.049) for the explicit measure of hand direction and 0.351 (0.026) for the implicit measure. Both of these values were less than the respective values of the alternated condition. A 2 (context: alternated vs randomized) x 2 (type of measure: hand-implicit vs hand-explicit) ANOVA revealed significant main effects both of type of measure ($F(1,44)=69.8$ $P<0.001$) and context ($F(1,44)=5.2$, $P<0.05$). The interaction was not significant ($F(1,44)=1.7$, $P>0.05$). Thus, there was no evidence of a different effect of global context on explicit and implicit measures of the bias of sensed hand direction toward the direction of the cursor.

Regarding the explicit measure of the bias of sensed cursor direction toward the direction of the hand, the mean (SE) of the individual biases in the blocked-cursor condition (-0.055±0.017) did not differ from that in the alternated condition (-0.077±0.014, $t(22)=1.36$, $P>0.05$). Similarly, the mean (SE) bias in the randomized condition (-0.076±0.017) did not differ from that in the alternated condition ($t(44)=0.04$, $P>0.05$). Thus, there was no indication that a stronger bias of hand judgments in one of the experimental conditions was accompanied by a weaker bias of cursor judgments.
